# Molecular imaging and biochemical response assessment after a single cycle of [^225^Ac]Ac-PSMA-617/[^177^Lu]Lu-PSMA-617 tandem therapy in mCRPC patients who have progressed on [^177^Lu]Lu-PSMA-617 monotherapy

**DOI:** 10.7150/thno.56211

**Published:** 2021-02-16

**Authors:** Florian Rosar, Fabian Hau, Mark Bartholomä, Stephan Maus, Tobias Stemler, Johannes Linxweiler, Samer Ezziddin, Fadi Khreish

**Affiliations:** 1 Department of Nuclear Medicine, Saarland University - Medical Center, Homburg, Germany; 2 Department of Urology, Saarland University - Medical Center, Homburg, Germany

**Keywords:** ^225^Ac and ^177^Lu, PSMA radioligand therapy, Biochemical response, Molecular imaging response, Metastatic castration-resistant prostate cancer.

## Abstract

**Rationale:** Despite the promising results of prostate-specific membrane antigen (PSMA)-targeted ^177^Lu radioligand therapy in metastatic castration-resistant prostate carcinoma (mCRPC), some patients do not respond and other patients with initially good response develop resistance to this treatment. In this study, we investigated molecular imaging and biochemical responses after a single cycle of [^225^Ac]Ac-PSMA-617/[^177^Lu]Lu-PSMA-617 tandem therapy in patients who had progressed on [^177^Lu]Lu-PSMA-617 monotherapy.

**Methods:** Seventeen patients with mCRPC were included in a retrospective, monocenter study. Molecular imaging-based response was assessed by modified PERCIST criteria using the whole-body total lesion PSMA (TLP) and molecular tumour volume (MTV) derived from [^68^Ga]Ga-PSMA-11 PET/CT. Biochemical response was evaluated according to PCWG3 criteria using the prostate-specific antigen (PSA) serum value. Concordance and correlation statistics as well as survival analyses were performed.

**Results:** Based on the molecular imaging-based response assessment, 5 (29.4%) patients showed partial remission and 7 (41.2%) had stable disease. The remaining 5 (29.4%) patients had further progression, four with an increase in TLP/MTV of >30% and one with stable TLP/MTV but appearance of new metastases. Based on the biochemical response assessment, 5 (29.4%), 8 (47.1%), and 4 (23.5%) patients showed partial remission, stable disease, and progressive disease, respectively. A comparison of the response assessment methods showed a concordance of 100% (17/17) between TLP and MTV and 70.6% (12/17) between TLP/MTV and PSA. Patients with partial remission, independently assessed by each method, had better overall survival (OS) than patients with either stable or progressive disease. The difference in OS was statistically significant for the molecular imaging response assessment (median OS not reached vs. 8.3 m, *p* = 0.044), but not for the biochemical response assessment (median OS 18.1 m vs. 9.4 m, *p* = 0.468).

**Conclusion:** Based on both assessment methods, [^225^Ac]Ac-PSMA-617/[^177^Lu]Lu-PSMA-617 tandem therapy is an effective treatment for the highly challenging cohort of patients with mCRPC who have progressed on [^177^Lu]Lu-PSMA-617 monotherapy. Molecular imaging response and biochemical PSA response were mostly concordant, though a considerable number of cases (29.4%) were discordant. Molecular imaging response reflecting the change in total viable tumour burden appears to be superior to PSA change in estimating survival outcome after tandem therapy.

## Introduction

Prostate carcinoma is the second most frequent malignancy in men 1, and a considerable number of patients ultimately progress to a metastatic castration-resistant stage (mCRPC) 2,3. Apart from chemotherapy with taxanes (docetaxel, cabazitaxel) and next-generation androgen receptor signal inhibitors (abiraterone, enzalutamide) 4-6, radioligand therapy targeting prostate-specific membrane antigen (PSMA) is a promising therapy option, currently applied after failure of chemotherapy and hormone therapy 7. PSMA radioligand therapy (PSMA-RLT) using PSMA ligands labelled with the beta emitter lutetium-177 (e.g. [^177^Lu]Lu-PSMA-617) has shown encouraging results in various retrospective studies 8-11 and in a few prospective clinical trials on patients with mCRPC 12,13. Some patients, however, do not respond to PSMA-RLT with beta emitters and other patients with initially good response develop resistance 14-16. This cohort of patients has limited remaining therapeutic options. Recently, PSMA-RLT with alpha emitters such as actinium-225 e.g. in form of [^225^Ac]Ac-PSMA-617 monotherapy 17-20 or [^225^Ac]Ac-PSMA-617/[^177^Lu]Lu-PSMA-617 tandem therapy, a concept first introduced by our group 21, has yielded impressive results. PSMA-RLT using alpha emitters may also be effective in patients who have progressed on [^177^Lu]Lu-PSMA-617 monotherapy due to the higher radiobiological impact of alpha radiation (20 times higher weighting factor than beta radiation) 22-24. However, the higher radiobiological impact of alpha radiation also affects 'organs-at-risk', especially the salivary glands, which may lead to severe xerostomia and impair patients' quality of life. Tandem therapy combining alpha and beta emitters with adapted doses, instead of monotherapy with alpha emitter, may reduce the occurrence of these substantial adverse effects. There is little data, however, about the efficacy of alpha emitters in these highly challenging patients. Besides biochemical response assessment, molecular imaging-based response assessment could be of high importance and interest but has not been reported. In this study, we investigated the efficacy of a single cycle of [^225^Ac]Ac-PSMA-617/[^177^Lu]Lu-PSMA-617 tandem therapy in patients who had progressed on [^177^Lu]Lu-PSMA-617 monotherapy. We assessed molecular imaging response by [^68^Ga]Ga-PSMA-11 positron emission tomography (PET)/computed tomography (CT) of total tumour burden and biochemical response by serum prostate-specific antigen (PSA) values. Both response assessment methods were evaluated as potential predictors of outcome for this tandem therapy approach.

## Methods

### Study design

**Figure 1:**
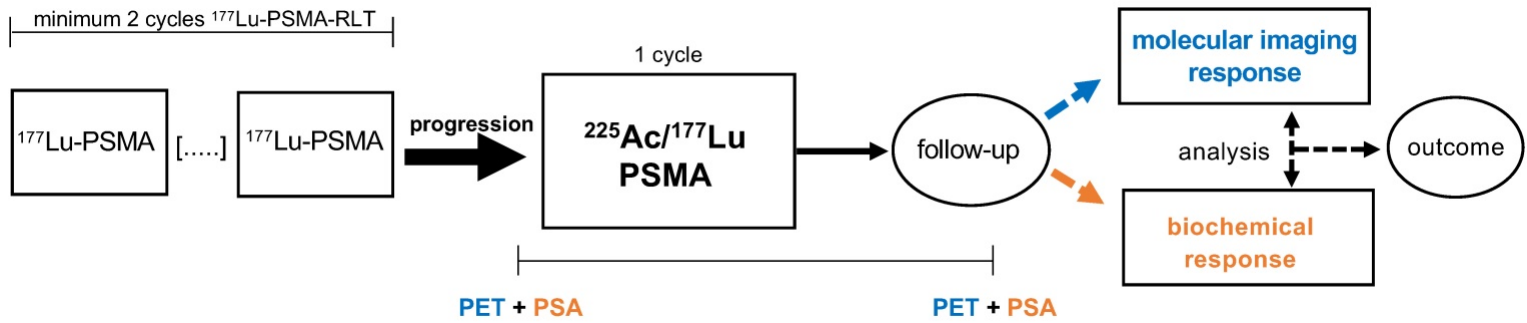
Study design. Patients who had progressed on at least 2 cycles [^177^Lu]Lu-PSMA-617 RLT were treated with 1 cycle [^225^Ac]Ac-PSMA-617/[^177^Lu]Lu-PSMA-617 tandem RLT. Molecular imaging response and biochemical response were evaluated by PET and PSA serum value in follow-up. Both response assessment methods were compared and analysed as potential predictors of outcome for this tandem therapy approach.

We performed a retrospective monocenter study of mCRPC patients treated with one cycle of [^225^Ac]Ac-PSMA-617/[^177^Lu]Lu-PSMA-617 tandem therapy after progression on [^177^Lu]Lu-PSMA-617 monotherapy. Progression of disease on [^177^Lu]Lu-PSMA-617 monotherapy was defined as a serum PSA increase of >25% in comparison to the previous cycle and an increased uptake (in target lesions) or appearance of new metastases by [^68^Ga]Ga-PSMA-11 PET/CT. Only patients with at least two cycles of [^177^Lu]Lu-PSMA-617 RLT were included to avoid the potential flare phenomenon of the initial cycle. Additionally, patients must have received [^68^Ga]Ga-PSMA-11 PET/CT within approximately one month before and another PET/CT scan within a few months after [^225^Ac]Ac-PSMA-617/[^177^Lu]Lu-PSMA-617 tandem therapy in order to assess response by molecular imaging. The study design is depicted schematically in **Figure 1**. Patients (n=6) who had progressed on [^177^Lu]Lu-PSMA-617 RLT but with known 'mismatch findings', meaning intense glucose metabolism on [^18^F]FDG PET/CT with missing or low PSMA expression, were excluded from the tandem therapy. In addition, patients with abnormal hematological blood parameters (hemoglobin < 8 mg/dL, platelets < 75,000 /μL, leukocytes < 3000 /μL), renal insufficiency (estimated glomerular filtration rate (eGFR) < 45 mL/min), or poor overall conditions (Eastern Cooperative Oncology Group (ECOG) performance status ≥ 3) did not receive tandem therapy.

### Patients and ethics

Seventeen mCRPC patients were included in this retrospective study. All patients received several pre-treatments. Detailed information about the pre-treatments and patient characteristics are presented in **Table 1**. The patients received an average of 5 ± 2 cycles (range: 2-9 cycles) of [^177^Lu]Lu-PSMA-617 monotherapy with a cumulative mean ^177^Lu activity of 35 ± 13 GBq (range: 14-66 GBq) before the [^225^Ac]Ac-PSMA-617/[^177^Lu]Lu-PSMA-617 tandem therapy cycle. The mean time from the start of [^177^Lu]Lu-PSMA-617 monotherapy was 40 ± 25 weeks (range: 11-111 weeks). The mean PSA increase from the last cycle of [^177^Lu]Lu-PSMA-617 monotherapy to [^225^Ac]Ac-PSMA-617/[^177^Lu]Lu-PSMA-617 tandem therapy was 278 ± 547% (range: 26-2338%). [^225^Ac]Ac-PSMA-617/[^177^Lu]Lu-PSMA-617 tandem therapy and [^68^Ga]Ga-PSMA-11 PET/CT were performed on a compassionate use basis under the German Pharmaceutical Act §13 (2b). Patients gave written consent after being informed in detail about the risks and potential side effects of these interventions. Patients consented additionally to publication of any resulting data in accordance with the Declaration of Helsinki. The study was approved by the local institutional review board (ethics committee permission number 140/17).

### [^225^Ac]Ac-PSMA-617/[^177^Lu]Lu-PSMA-617 tandem therapy

[^225^Ac]Ac-PSMA-617 and [^177^Lu]Lu-PSMA-617 were synthesized according to published procedures described in detail by Kratochwil et al. 14,25. Both radioligands were administered simultaneously during an inpatient stay in accordance with German radiation protection regulations. The mean administered activity of [^225^Ac]Ac-PSMA-617 and [^177^Lu]Lu-PSMA-617 was 4 ± 2 MBq (range: 1.8-6.9 MBq), corresponding to 44 ± 18 kBq/kg body weight (BW) (range: 19-74 kBq/kg BW), and 6 ± 1 GBq (range: 3.8-8.2 GBq), respectively. Administered activities of ^225^Ac and ^177^Lu were primarily chosen with regard to total tumour burden, sites of metastases, and patient condition including ECOG performance status, organ function, and degree of existing xerostomia before tandem therapy. For example, patients with high tumour burden or patients who never responded to [^177^Lu]Lu-PSMA-617 monotherapy received higher ^225^Ac activities with high ^225^Ac/^177^Lu ratios (up to a ratio of 1 MBq ^225^Ac to 1 GBq ^177^Lu). Patients with low tumour burden or unfavorable patient conditions received lower total amounts of ^225^Ac and/or lower ^225^Ac/^177^Lu ratios. In order to prevent side effects, each patient received intravenous hydration (1000 mL 0.9% NaCl) 30 min before to 120 min after injection of the radioligand and cooling of the salivary glands.

### [^68^Ga]Ga-PSMA-11 PET/CT

Each patient was imaged by [^68^Ga]Ga-PSMA-11 PET/CT on average 5 ± 8 d (range: 1-35 d) prior to and 53 ± 32 d (range: 21-119 d) after tandem therapy. A mean activity of 124 ± 17 MBq (range: 83-155 MBq) [^68^Ga]Ga-PSMA-11 was administered intravenously, followed by a 500 mL infusion of 0.9% NaCl. No diuretics were applied. Before administering the radioactivity, blood tests including PSA serum value, creatinine, hemoglobin, leukocytes, and platelets were performed. The mean time between injection and PET acquisition was ~60 min, in accordance with standard procedures for prostate cancer imaging 26. The PET/CT scans were performed using a Biograph 40 mCT PET/CT (Siemens Medical Solutions, Knoxville, TN, USA) accredited by European Association of Nuclear Medicine (EANM) Research Ltd. PET acquisition was performed from vertex to mid-femur with an acquisition time of 3 min per bed position, which covers a 21.4 cm extended field-of-view (TrueV). The PET datasets were reconstructed using an iterative 3-dimensional ordered subset expectation maximization algorithm (3 iterations; 24 subsets) with gaussian filtering and a slice thickness of 5 mm. Random correction, decay correction, scatter correction, and attenuation correction were applied. CT was performed with a low-dose technique using an X-ray tube voltage of 120 keV and tube current modulated by CARE Dose4D with a maximal tube current-time product of 30 mAs.

### Response assessment

**Figure 2:**
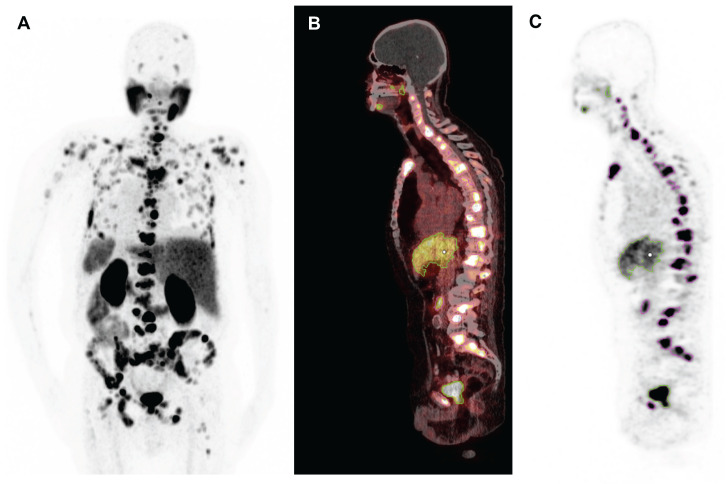
Representative example of semi-automatic tumour segmentation using Syngo.Via. **(A)** Maximum intensity projection of [^68^Ga]Ga-PSMA-11 PET/CT. **(B)** Sagittal slice of PET/CT fusion. **(C)** PET data with semi-automatically drawn volumes of interest (violet, tumour lesions; green, physiological uptake).

Molecular imaging-based response was assessed by calculating the whole-body total lesion PSMA (TLP) and molecular tumour volume (MTV) from [^68^Ga]Ga-PSMA-11 PET/CT collected before and after [^225^Ac]Ac-PSMA-617/[^177^Lu]Lu-PSMA-617 tandem therapy by applying a semi-automatic tumour segmentation using Syngo.Via (Enterprise VB 30, Siemens, Erlangen, Germany). For tumour segmentation, a standard uptake value (SUV) threshold of 3.0 was applied, in accordance with Ferdinandus et al. 27, except for liver metastases (in 2/17 patients), where an SUV threshold of 1.5 × SUV_mean_ of the healthy liver tissue was applied. Physiological uptake in healthy organs was manually excluded. A representative example of the semi-automatic tumour segmentation is presented in **Figure 2**. TLP was calculated in analogy to total lesion glycolysis (TLG), which is an established parameter in [^18^F]FDG PET/CT for therapy monitoring 28, as the summed products of volume and uptake (SUV_mean_) of all lesions. TLP values are expressed as mL × SUV to distinguish them from MTV values, which are presented in mL. Between PET scans, androgen deprivation therapy (ADT) and therapy with next-generation androgen receptor signal inhibitors such as enzalutamide or abiraterone were continued unchanged to avoid altering PSMA expression 29 (ADT in 17/17, enzalutamide in 13/17, and abiraterone in 1/17 patients). As in other studies 30,31, the PET response criteria in solid tumours (PERCIST) version 1.0 32 were slightly modified: partial remission was defined as a decrease in TLP or MTV of >30%, progression as an increase of >30% or development of new metastases, and stable disease as a change between ±30%.

Biochemical response was evaluated by the serum PSA value measured on the same day that the PET/CT scans were collected using the Prostate Cancer Working Group 3 criteria (PCWG3) 33: partial remission was defined as a decrease in PSA of >50%, progression as an increase in PSA of >25%, and stable disease as a change between -50% and +25%. However, these changes were not confirmed by a second measurement as is recommended by the PCWG3.

### Toxicity

Hematotoxicity, renal toxicity, and xerostomia were assessed after one cycle of [^225^Ac]Ac-PSMA-617/[^177^Lu]Lu-PSMA-617 tandem therapy. Toxicity was recorded using the Common Terminology Criteria for Adverse Events version 5.0 (CTCAE) on blood values of hemoglobin, leucocytes, and platelets, and eGFR. CTCAE grades of xerostomia were assessed based on patient reporting via a questionnaire.

### Statistics and survival analysis

Descriptive and concordance analyses were performed on the molecular imaging (TLP and MTV) and biochemical (PSA) response assessments. In addition, several parameters such as patient characteristics, imaging parameters, pretherapeutic parameters, and therapeutic parameters were tested for correlation with changes in TLP, MTV, and PSA. Spearman's correlation was applied for this purpose using Prism 8 (GraphPad Software, San Diego, USA). A *p*-value of <0.05 was regarded as statistically significant. Overall survival (OS) was defined as the interval from the start of [^225^Ac]Ac-PSMA-617/[^177^Lu]Lu-PSMA-617 tandem therapy to the occurrence of any of the following: (1) death from any cause, (2) commencement of a different treatment such as chemotherapy, or (3) the last study visit. Patients were independently categorized by molecular imaging and biochemical response assessments into two groups: (a) patients with partial remission and (b) patients with stable or progressive disease. The cut-off follow-up date was October 30^th^, 2020. Median follow-up was determined, and OS was analyzed using the Kaplan-Meier method.

## Results

Prior to [^225^Ac]Ac-PSMA-617/[^177^Lu]Lu-PSMA-617 tandem therapy, the median baseline values of TLP, MTV, and PSA were 3685 mL × SUV (range: 723-13679 mL × SUV), 453 mL (range: 153-2581 mL), and 152 ng/mL (range: 5.9-2570 ng/mL), respectively. There was no significant correlation between baseline TLP and PSA (*r* = 0.03, *p* = 0.90) or MTV and PSA (*r* = 0.11, *p* = 0.67), but there was a significantly high correlation between TLP and MTV (*r* = 0.99, *p* < 0.01). After one cycle of tandem therapy, the median values of TLP, MTV, and PSA were 2289 mL × SUV (range: 72-35819 mL × SUV), 421 mL (range: 14-4542 mL), and 135 ng/mL (range: 6.1-1251 ng/mL), respectively. The TLP, MTV, and PSA values for each patient are compiled in **Table 2**.

### Molecular imaging response

**Figure 3:**
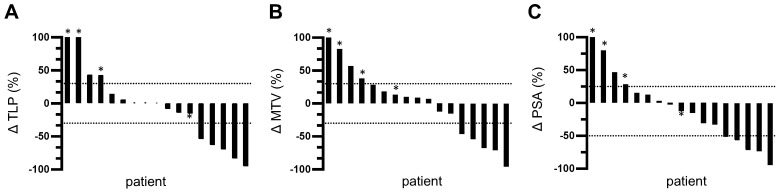
Waterfall plots of relative changes in **(A)** TLP, **(B)** MTV, and **(C)** PSA. * indicates appearance of new metastases. Values over 100% were cropped for simplification.

TLP and MTV responses after one cycle of tandem therapy were similar for each patient. 5/17 patients (29.4%) showed partial remission with a decrease in TLP/MTV of >30%. None of these patients developed new metastases. 8/17 patients showed a change in TLP and MTV between -30% and +30%. However, one of these patients (5.9%) developed new metastases and was classified as having progressive disease. Thus, stable disease was found in 41.2% of patients (7/17). The remaining 4 patients (23.5%) showed disease progression with an increase in TLP/MTV of >30% and three of these patients developed new metastases. The changes in TLP and MTV are visualized as waterfall plots in **Figure 3A-B**.

### Biochemical response

5/17 patients (29.4%) showed partial remission based on a decrease in PSA of >50%. 8/17 patients (47.1%) exhibited stable disease with a PSA change between -50% and +25%. The remaining 4/17 patients (23.5%) showed disease progression with an increase in PSA of >25%. A waterfall plot of PSA changes is depicted in **Figure 3C**.

### Concordance analyses

**Figure 4:**
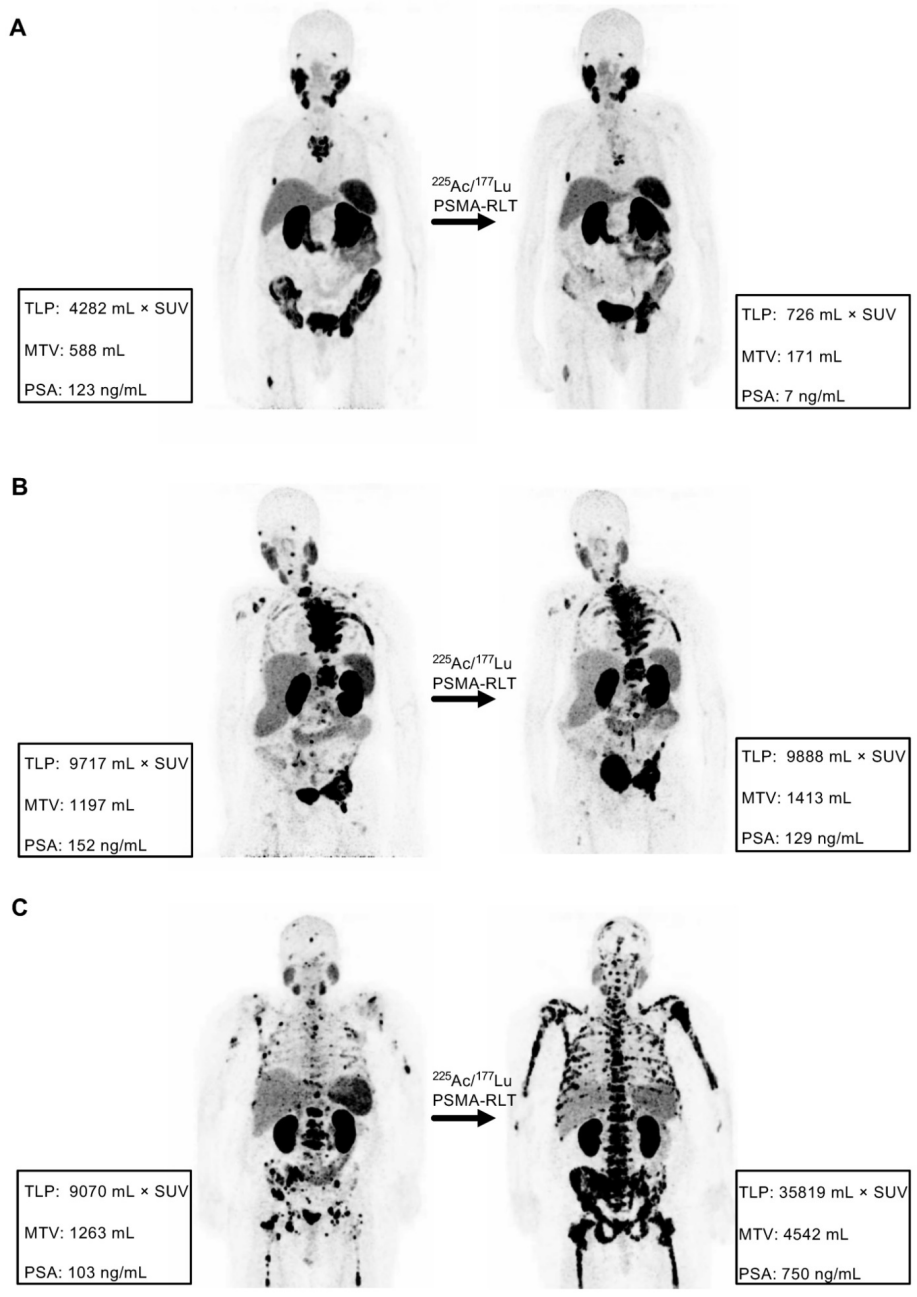
Examples of concordance between molecular imaging response assessment (TLP, MTV) and biochemical response assessment (PSA). **(A)** Partial remission in patient #8 (TLP -83%, MTV -71%, PSA -94%). **(B)** Stable disease in patient #4 (TLP +2%, MTV +18%, PSA -15%). **(C)** Progressive disease in patient #7 (TLP +295%, MTV +260%, PSA +628%).

**Figure 5:**
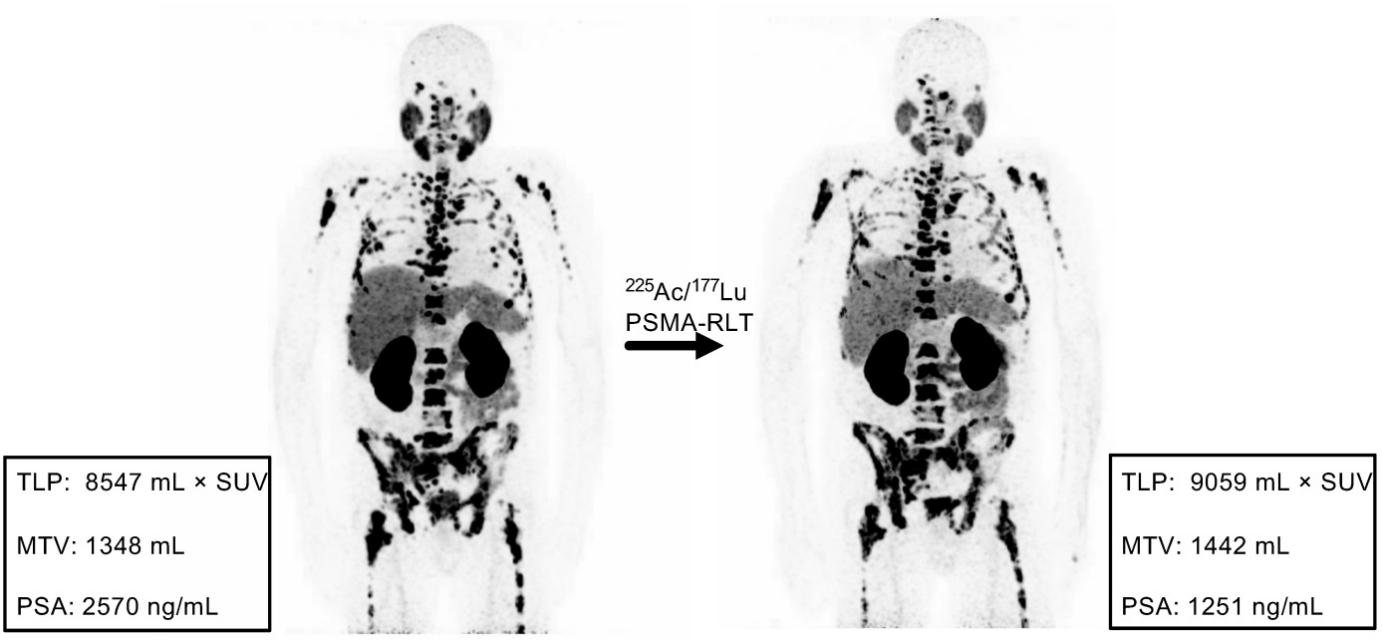
Example of discordance between molecular imaging response assessment (TLP, MTV) and biochemical response assessment (PSA). Patient #11 shows stable disease by TLP (+6%) and MTV (+7%) and partial remission by PSA (-51%).

Comparison of the response assessment methods showed a concordance of 100% (17/17) between TLP and MTV and 70.6% (12/17) between TLP/MTV and PSA (**Table 3**). Concordant examples of partial remission, stable disease, and progression are shown in **Figure 4**. Of the discordant patients, two patients had stable disease by molecular imaging but one had partial remission and one had progressive disease by PSA, one patient had partial remission by molecular imaging and stable disease by PSA, and two patients had progressive disease according to molecular imaging but stable disease by PSA (**Table 3**). A discordant example is presented in **Figure 5**.

### Correlation with pre-therapeutic parameters

Multiple pre-therapeutic parameters were tested for correlation with changes in TLP, MTV, and PSA. All pre-therapeutic parameters tested with their corresponding *r*- and *p*-values are summarized in **Table 4**. No significant correlation was noted for any of the parameters tested.

### Toxicity

[^225^Ac]Ac-PSMA-617 and [^177^Lu]Lu-PSMA-617 were well-tolerated without any serious acute adverse events. No relevant change in eGFR was observed after the single cycle of tandem therapy. One patient had grade 3 thrombocytopenia but no other grade 3/4 hematotoxicities were observed. One patient experienced mild (grade 1) xerostomia attributed to the tandem therapy. In 4 patients with known mild xerostomia prior to tandem therapy, no change in xerostomia grade was recorded. Detailed CTCAE prior to and after tandem therapy are summarized in **Table 5**. In patients with stable disease or partial remission by both assessment methods, no relevant weight loss and no decrease in overall ECOG performance status was observed. Rather, reduction in pain, as assessed by the visual analogue scale, was noted in 3 patients.

### Survival analysis

**Figure 6:**
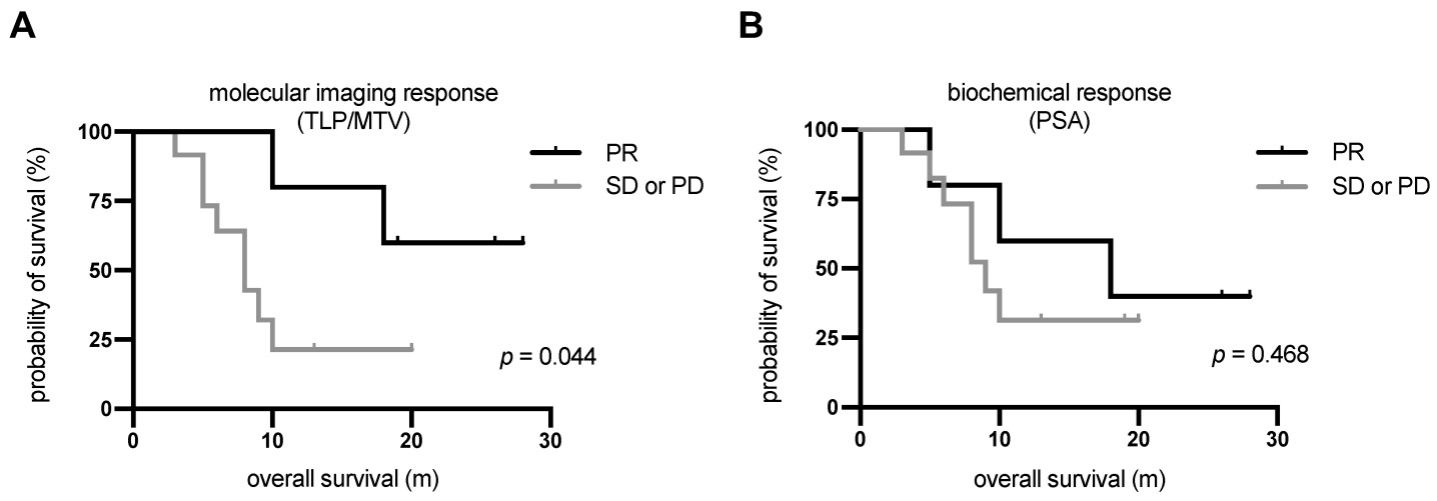
Kaplan-Meier curves for OS stratified by **(A)** molecular imaging response assessment and **(B)** biochemical response assessment. PD, progressive disease; PR, partial remission; SD, stable disease.

The median follow-up time was 20 m (range: 3-28 m) from the date of [^225^Ac]Ac-PSMA-617/[^177^Lu]Lu-PSMA-617 tandem therapy. All patients showed disease progression in the follow-up time. The median PSA-based progression-free survival (PFS) was 3.7 m (95%CI: 3.0-4.4 m). Ten patients (58.8%) died by the end of the study. All deaths were mCRPC related. Patients showing partial remission by molecular imaging after one cycle of tandem therapy had not yet reached the median OS after a median follow-up time of 20 m. The median OS of patients showing either stable or progressive disease according to molecular imaging was 8.3 m (95%CI: 4.8-11.7 m). The difference in median OS between these patients and those showing partial remission was statistically significant (*p* = 0.044, log-rank test). The corresponding Kaplan-Meier curves are shown in **Figure 6A**. In contrast, there was no significant difference (*p* = 0.468) in OS between patients showing a PSA decline of >50% (median: 18.1 m, 95%CI: 1.1-35.1 m) and those with biochemically stable or progressive disease (median: 9.4 m, 95%CI: 7.3-11.4 m). The corresponding Kaplan-Meier curves are shown in **Figure 6B**. The 3 patients who showed disease progression by both response assessment methods did not receive further radioligand therapy. The remaining 14 patients continued radioligand therapy: 5 received [^225^Ac]Ac-PSMA-617/[^177^Lu]Lu-PSMA-617 tandem therapy cycles, 4 received [^177^Lu]Lu-PSMA-617 therapy cycles, and 5 received both.

## Discussion

This is the first study investigating molecular imaging and biochemical response assessments after a single cycle of [^225^Ac]Ac-PSMA-617/[^177^Lu]Lu-PSMA-617 tandem therapy in patients with mCRPC (*n* = 17) who had progressed on [^177^Lu]Lu-PSMA-617 monotherapy. In this highly challenging patient cohort, 29.4% (5/17) of patients showed partial remission by either response assessment method after the single cycle of tandem therapy. Our study shows that a PSMA-targeted tandem therapy with alpha and beta emitters can be a successful treatment option for patients with mCRPC and resistance to monotherapy with beta emitters. The results are also in agreement with our previous study 21 in a different cohort of patients who had an insufficient response to monotherapy with [^177^Lu]Lu-PSMA-617. In the present study, the patients had progressive disease by both [^68^Ga]Ga-PSMA-11 PET/CT and serum PSA (PSA increase of >25% since last cycle), whereas patients in our previous study were only insufficient responders (defined by a PSA increase or decrease of <50%). These different inclusion criteria may explain why the PSA-based response rate after a median follow-up time of 2 m in this study is lower than that of the previous study (29.4% vs. 50%). All patients were heavily pre-treated with at least one line taxane (if not contraindicated) and next-generation androgen receptor signal inhibitors (enzalutamide and abiraterone), and showed resistance to [^177^Lu]Lu-PSMA-617 monotherapy. These patients, accordingly, had limited therapeutic options remaining.

Two different methods, molecular imaging assessment using [^68^Ga]Ga-PSMA-11 PET/CT to calculate TLP and MTV and biochemical assessment of serum PSA, were used to evaluate response. Comparing the TLP and MTV values before tandem therapy, a high correlation (*r* = 0.99 with *p* < 0.01) was observed as both reflect the total tumour burden. In assessing response, TLP and MTV provided a concordance of 100%, which is in line with the results of Hartrampf et al. 34*.* No correlation between pre-therapeutic TLP or MTV and PSA was noted in our cohort (*r* = 0.03 with *p* = 0.90 and *r* = 0.11 with *p* = 0.67, respectively). Therefore, molecular imaging and PSA can be considered independent response assessment methods. For most patients, molecular imaging response was in accordance with biochemical response, which is currently the standard response method used in the clinic. A concordance of 70.6% (12/17) between molecular imaging and biochemical methods was noted in our study. Similar concordances of 65-87% have been observed in other studies with different cohorts of patients with prostate cancer 35-37. Since 29.4% of patients had discordant results, a survival analysis was performed to evaluate which of these two methods better estimates the therapy outcome. Patients with partial remission, independently assessed by each method, had better OS than patients with either stable or progressive disease. The difference in OS was statistically significant for the molecular imaging response assessment (median OS not reached vs. 8.3 m, *p* = 0.044), but not for the biochemical response assessment (median OS 18.1 m vs. 9.4 m, *p* = 0.468). Accordingly, molecular imaging response can be considered a prognostic value for outcome of this tandem therapy. Despite the very small number of patients, this preliminary data suggests the need for treatment monitoring by [^68^Ga]Ga-PSMA-11 PET/CT in patients with mCRPC undergoing tandem RLT. Based on our results, the total tumour burden by [^68^Ga]Ga-PSMA-11 PET/CT (measured as either TLP or MTV) is an important parameter for monitoring PSMA-targeted tandem RLT and may be used to assess other treatments in patients with mCRPC. To confirm our findings, future studies in larger patient cohorts are recommended. No significant correlation was noted between several pre-therapeutic parameters and changes in TLP, MTV, or PSA (**Table 4**). No patient, therefore, should be excluded from tandem RLT based on any of these parameters. The statistical analysis may be biased by the small number of patients and further studies are needed.

One cycle of [^225^Ac]Ac-PSMA-617/[^177^Lu]Lu-PSMA-617 tandem therapy was safe and well-tolerated. The patient, who experienced grade 3 thrombocytopenia had extensive progression with disseminated bone metastases (the patient is depicted in **Figure 4C**). This thrombocytopenia seemed to be more related to progression of disease than to the tandem therapy. No other grade 3/4 hematotoxicities were observed. Only one patient experienced mild xerostomia attributed to the cycle of tandem therapy. This low rate of toxicity, especially of xerostomia, may be related to the lower administered activity of ^225^Ac (mean: 44 kBq/kg BW) in comparison to other studies applying [^225^Ac]Ac-PSMA-617 as monotherapy 19,20,22. For example, Kratochwil et al. recommended 100 kBq/kg BW in [^225^Ac]Ac-PSMA-617 monotherapy to balance response and side effects 17. Besides the expected low toxicity profile of the tandem therapy in comparison to ^225^Ac monotherapy, we also assume, as discussed in our previous study 21, that ^177^Lu may still have a contributing anti-tumour effect when augmented with low activities of ^225^Ac in patients somewhat resistant to ^177^Lu monotherapy. Alpha radiation even in lower quantities might be able to overcome beta resistance. From our clinical experience, this seems to be true to some extent and was observed in 4/9 patients that were re-challenged [^177^Lu]Lu-PSMA-617 therapy after tandem therapy (1 patient with partial remission, 3 patients with stable disease); however, this hypothesis remains completely unproven.

Our results provide preliminary evidence and a rational starting point for further studies. Ideally, prospective studies should be conducted to establish the tandem approach in mCRPC management. A dose-finding study that includes evaluation of the absolute activities of ^225^Ac and ^177^Lu as well as the ^225^Ac/^177^Lu ratio may be one of the next steps to improve response without impairing quality of life. In addition, evaluation of tandem therapy is also conceivable in patients without resistance to ^177^Lu monotherapy.

### Limitations

The promising results reported herein should be considered in the light of some limitations. The study suffers from its retrospective nature and the limited number of patients. A further limitation is that the time for follow-up varied, which could impact the TLP, MTV, and PSA changes. The majority of patients received their follow-up ([^68^Ga]Ga-PSMA-11 PET/CT and serum PSA on the same day) about 4 to 8 weeks after the tandem therapy. In a few patients, this time window could not be met due to the patient's condition or organizational reasons. In these few outliers, however, the assessment methods showed concordant results and did not impact the survival analyses. Another major limitation of this study is that no fixed activity protocol was used. The ^225^Ac and ^177^Lu activities were individually chosen based on tumour and patient characteristics. Furthermore, the limited availability of ^225^Ac influenced the determination of the administered activities, resulting in a variable ^225^Ac/^177^Lu ratio. In addition, it has to be pointed out that there are several methods for calculating TLP and MTV 27,35,36. Even though percentage-based thresholding (41% or 50%) is recommended by the EANM for TLG in [^18^F]FDG-PET/CT 28, this method is known to be adequate solely for non-heterogenous uptake distributions. In order to not underestimate the lesion volume in case of heterogeneous PSMA expression, which is often observed in disseminated and confluent disease after many therapies, we applied the method published by Ferdinandus et al. 27 with a fixed SUV threshold of 3.0. For calculating liver metastases, we applied a threshold of 1.5 × SUV_mean_ of the healthy liver, which appeared to be appropriate compared to visual findings.

## Conclusion

[^225^Ac]Ac-PSMA-617/[^177^Lu]Lu-PSMA-617 tandem therapy is an effective treatment for the highly challenging cohort of patients with mCRPC who have progressed on [^177^Lu]Lu-PSMA-617 monotherapy. Molecular imaging response (based on the [^68^Ga]Ga-PSMA-11 PET/CT-derived parameters TLP and MTV) and biochemical serum PSA response were mostly concordant, though a considerable number of cases (29.4%) were discordant. Molecular imaging response reflecting the change in viable total tumour burden appears to be superior to PSA change in estimating survival outcome after tandem therapy. Larger, and ideally prospective, studies are recommended to confirm and expand these findings.

## Figures and Tables

**Table 1:** Patient characteristics.

Characteristics	
Age (y)	69.4 ± 8.3 (57.0-89.0)
ECOG performance status	
0	5 (29.4%)
1	11 (64.7%)
2	1 (5.9%)
Previous treatments	
Prostatectomy	12 (70.6%)
Radiation	12 (70.6%)
ADT	17 (100%)
Enzalutamide or Abiraterone	17 (100%)
Enzalutamide	13 (76.5%)
Abiraterone	15 (88.2%)
Enzalutamide and Abiraterone	11 (64.7%)
Chemotherapy	13 (76.5%)
Docetaxel	13 (76.5%)
Cabazitaxel	7 (41.2%)
Docetaxel and Cabazitaxel	7 (41.2%)
^223^Ra therapy	5 (29.4%)
[^177^Lu]Lu-PSMA-617 RLT	17 (100%)
Cycles	5 ± 2 (2-9)
Cumulative ^177^Lu activity (GBq)	35.3 ± 13.3 (13.7-66.1)
**Sites of metastases**	
Bone	17 (100%)
Lymph node	5 (29.4%)
Liver	2 (11.8%)
Other	1 (5.9%)

**Table 2:** Absolute values of TLP, MTV, and PSA prior to and after tandem therapy (values over 15 were rounded to full values).

Patient number	TLP prior to therapy (mL × SUV)	TLP after therapy (mL × SUV)	MTV prior to therapy (mL)	MTV after therapy (mL)	PSA prior to therapy (ng/mL)	PSA after therapy (ng/mL)
**1**	934	1874	187	342	191	344
**2**	13679	5113	2581	1184	1277	367
**3**	5412	7750	838	1159	60	77
**4**	9717	9888	1197	1413	152	129
**5**	4290	4363	617	672	258	379
**6**	4365	2026	784	421	10.1	6.8
**7**	9070	35819	1263	4542	103	750
**8**	4282	726	588	171	123	7.0
**9**	1448	72	303	13	98	26
**10**	723	664	153	130	251	290
**11**	8547	9059	1348	1442	2570	1251
**12**	2142	2446	372	477	508	496
**13**	3685	3123	452	513	115	101
**14**	1447	1242	259	228	13.0	9.0
**15**	1959	596	368	119	311	135
**16**	1153	1166	265	293	968	1091
**17**	1594	2289	214	335	5.9	6.1

**Table 3:** Changes in TLP, MTV, and PSA and their respective responses. PD, progressive disease; PR, partial remission; SD, stable disease. * indicates patients with new metastases by [^68^Ga]Ga-PSMA-11 PET/CT. Patients with discordant molecular imaging and biochemical responses are underlined.

Patient number	ΔTLP (%)	ΔMTV (%)	ΔPSA (%)	TLP response	MTV response	PSA response
**1**	100.7*	82.7*	80.1	PD	PD	PD
**2**	-62.6	-54.1	-71.3	PR	PR	PR
**3**	43.2*	38.3*	28.3	PD	PD	PD
**4**	1.8	18.1	-15.1	SD	SD	SD
**5**	1.7	8.9	46.9	SD	SD	PD
**6**	-53.6	-46.2	-32.7	PR	PR	SD
**7**	294.9*	259.8*	628.2	PD	PD	PD
**8**	-83.0	-70.9	-94.3	PR	PR	PR
**9**	-95.1	-95.5	-73.5	PR	PR	PR
**10**	-8.1	-15.0	15.5	SD	SD	SD
**11**	6.0	7.0	-51.3	SD	SD	PR
**12**	14.2	28.3	-2.4	SD	SD	SD
**13**	-15.3*	13.3*	-12.2	PD	PD	SD
**14**	-14.1	-12.0	-30.8	SD	SD	SD
**15**	-69.6	-67.6	-56.6	PR	PR	PR
**16**	1.1	10.3	12.7	SD	SD	SD
**17**	43.6	56.8	3.2	PD	PD	SD

**Table 4:** Correlation between pre-therapeutic parameters and changes in TLP, MTV, and PSA. PFS, progression-free survival.

	ΔTLP		ΔMTV		ΔPSA	
	*r*	*p*	*r*	*p*	*r*	*p*
PSA prior to tandem therapy	-0.05	0.86	-0.15	0.56	-0.08	0.75
PSA increase from previous cycle prior to tandem therapy	0.08	0.75	-0.04	0.89	-0.25	0.32
SUV_max_ of the most intense lesion prior to tandem therapy	0.12	0.65	0.21	0.42	0.10	0.69
SUV_peak_ of the most intense lesion prior to tandem therapy	0.13	0.62	0.20	0.44	0.11	0.67
TLP tumour load prior to tandem therapy	0.08	0.77	0.06	0.81	-0.17	0.51
MTV tumour load prior to tandem therapy	0.02	0.95	-0.01	0.96	-0.20	0.43
Administered ^225^Ac activity	0.12	0.65	0.11	0.67	-0.21	0.41
Administered ^177^Lu activity	-0.09	0.72	-0.14	0.58	-0.22	0.40
Applied ^225^Ac/^177^Lu activity ratio	0.18	0.49	0.20	0.45	-0.05	0.86
Cumulative ^177^Lu activity prior to tandem therapy	-0.10	0.71	-0.21	0.43	-0.12	0.66
Time from initial diagnosis to tandem therapy	0.03	0.90	0.19	0.47	0.26	0.31
Time from initial diagnosis to castration resistance	0.00	0.10	0.13	0.61	0.20	0.44
PFS after chemotherapy (13/17 patients)	-0.26	0.40	-0.31	0.30	-0.16	0.61
Time from start of ^177^Lu-PSMA-RLT to tandem therapy	-0.43	0.09	-0.44	0.08	-0.24	0.36

**Table 5:** CTCAE before and after a single cycle of tandem therapy

	Before tandem therapy					After a single cycle tandem therapy				
	None	CTC 1°	CTC 2°	CTC 3°	CTC 4°	None	CTC 1°	CTC 2°	CTC 3°	CTC 4°
Xerostomia	13 (76.5%)	4 (23.5%)	0 (0%)	0 (0%)	0 (0%)	12 (70.6%)	5 (29.4%)	0 (0%)	0 (0%)	0 (0%)
eGFR	10 (58.8%)	6 (35.3%)	1 (5.9%)	0 (0%)	0 (0%)	10 (58.8%)	6 (35.3%)	1 (5.9%)	0 (0%)	0 (0%)
Hemoglobin	0 (0%)	15 (88.2%)	2 (11.8%)	0 (0%)	0 (0%)	0 (0%)	12 (70.6%)	5 (29.4%)	0 (0%)	0 (0%)
Leucocytes	11 (64.7%)	3 (17.6%)	3 (17.6%)	0 (0%)	0 (0%)	12 (70.6%)	3 (17.6%)	2 (11.8%)	0 (0%)	0 (0%)
Platelets	14 (82.4%)	3 (17.6%)	0 (0%)	0 (0%)	0 (0%)	14 (82.4%)	2 (11.8%)	0 (0%)	1 (5.9%)	0 (0%)

## Abbreviations

BW: body weight
CT: computed tomography
EANM: European Association of Nuclear Medicine
ECOG: Eastern Cooperative Oncology Group
eGFR: estimated glomerular filtration rate
mCRPC: metastatic castration-resistant prostate carcinoma
MTV: molecular tumour volume
PD: progressive disease
PET: positron emission tomography
PR: partial remission
PSA: prostate-specific antigen
PSMA: prostate-specific membrane antigen
PSMA-RLT: PSMA-targeted radioligand therapy
RLT: radioligand therapy
SD: stable disease
TLG: total lesion glycolysis
TLP: total lesion PSMA

## References

[1] Bray F, Ferlay J, Soerjomataram I, Siegel RL, Torre LA, Jemal A (2018). Global cancer statistics 2018: GLOBOCAN estimates of incidence and mortality worldwide for 36 cancers in 185 countries. CA Cancer J Clin.

[2] Kirby M, Hirst C, Crawford ED (2011). Characterising the castration-resistant prostate cancer population: a systematic review: The Epidemiology of CRPC. Int J Clin Pract.

[3] Watson PA, Arora VK, Sawyers CL (2015). Emerging mechanisms of resistance to androgen receptor inhibitors in prostate cancer. Nat Rev Cancer.

[4] Tannock IF, Horti J, Oudard S, James ND, Rosenthal MA (2004). Docetaxel plus Prednisone or Mitoxantrone plus Prednisone for Advanced Prostate Cancer. N Engl J Med.

[5] Berthold DR, Pond GR, Soban F, de Wit R, Eisenberger M, Tannock IF (2008). Docetaxel Plus Prednisone or Mitoxantrone Plus Prednisone for Advanced Prostate Cancer: Updated Survival in the TAX 327 Study. J Clin Oncol.

[6] Cassinello J, Carballido Rodríguez J, Antón Aparicio L (2016). Role of taxanes in advanced prostate cancer. Clin Transl Oncol.

[7] Kratochwil C, Fendler WP, Eiber M (2019). EANM procedure guidelines for radionuclide therapy with ^177^Lu-labelled PSMA-ligands (^177^Lu-PSMA-RLT). Eur J Nucl Med Mol Imaging.

[8] Baum RP, Kulkarni HR, Schuchardt C (2016). ^177^Lu-Labeled Prostate-Specific Membrane Antigen Radioligand Therapy of Metastatic Castration-Resistant Prostate Cancer: Safety and Efficacy. J Nucl Med.

[9] Rahbar K, Ahmadzadehfar H, Kratochwil C (2017). German Multicenter Study Investigating ^177^Lu-PSMA-617 Radioligand Therapy in Advanced Prostate Cancer Patients. J Nucl Med.

[10] Ahmadzadehfar H, Rahbar K, Baum RP (2020). Prior therapies as prognostic factors of overall survival in metastatic castration-resistant prostate cancer patients treated with [^177^Lu]Lu-PSMA-617. A WARMTH multicenter study (the 617 trial). Eur J Nucl Med Mol Imaging.

[11] Barber TW, Singh A, Kulkarni HR, Niepsch K, Billah B, Baum RP (2019). Clinical Outcomes of ^177^Lu-PSMA Radioligand Therapy in Earlier and Later Phases of Metastatic Castration-Resistant Prostate Cancer Grouped by Previous Taxane Chemotherapy. J Nucl Med.

[12] Hofman MS, Violet J, Hicks RJ (2018). [^177^Lu]-PSMA-617 radionuclide treatment in patients with metastatic castration-resistant prostate cancer (LuPSMA trial): a single-centre, single-arm, phase 2 study. Lancet Oncol.

[13] Emmett L, Crumbaker M, Ho B (2019). Results of a Prospective Phase 2 Pilot Trial of ^177^Lu-PSMA-617 Therapy for Metastatic Castration-Resistant Prostate Cancer Including Imaging Predictors of Treatment Response and Patterns of Progression. Clin Genitourin Cancer.

[14] Kratochwil C, Giesel FL, Stefanova M (2016). PSMA-Targeted Radionuclide Therapy of Metastatic Castration-Resistant Prostate Cancer with ^177^Lu-Labeled PSMA-617. J Nucl Med.

[15] Yadav MP, Ballal S, Sahoo RK, Dwivedi SN, Bal C (2019). Radioligand Therapy With ^177^Lu-PSMA for Metastatic Castration-Resistant Prostate Cancer: A Systematic Review and Meta-Analysis. AJR Am J Roentgenol.

[16] Rahbar K, Bode A, Weckesser M (2016). Radioligand Therapy With ^177^Lu-PSMA-617 as A Novel Therapeutic Option in Patients With Metastatic Castration Resistant Prostate Cancer. Clin Nucl Med.

[17] Kratochwil C, Bruchertseifer F, Rathke H (2017). Targeted α-Therapy of Metastatic Castration-Resistant Prostate Cancer with ^225^Ac-PSMA-617: Dosimetry Estimate and Empiric Dose Finding. J Nucl Med.

[18] Kratochwil C, Bruchertseifer F, Rathke H (2018). Targeted α-Therapy of Metastatic Castration-Resistant Prostate Cancer with ^225^Ac-PSMA-617: Swimmer-Plot Analysis Suggests Efficacy Regarding Duration of Tumor Control. J Nucl Med.

[19] Sathekge M, Bruchertseifer F, Knoesen O (2019). ^225^Ac-PSMA-617 in chemotherapy-naive patients with advanced prostate cancer: a pilot study. Eur J Nucl Med Mol Imaging.

[20] Sathekge M, Bruchertseifer F, Vorster M (2020). Predictors of Overall and Disease-Free Survival in Metastatic Castration-Resistant Prostate Cancer Patients Receiving ^225^Ac-PSMA-617 Radioligand Therapy. J Nucl Med.

[21] Khreish F, Ebert N, Ries M (2020). ^225^Ac-PSMA-617/^177^Lu-PSMA-617 tandem therapy of metastatic castration-resistant prostate cancer: pilot experience. Eur J Nucl Med Mol Imaging.

[22] Yadav MP, Ballal S, Sahoo RK, Tripathi M, Seth A, Bal C (2020). Efficacy and safety of ^225^Ac-PSMA-617 targeted alpha therapy in metastatic castration-resistant Prostate Cancer patients. Theranostics.

[23] Ilhan H, Gosewisch A, Böning G (2020). Response to ^225^Ac-PSMA-I&T after failure of long-term ^177^Lu-PSMA RLT in mCRPC. Eur J Nucl Med Mol Imaging.

[24] Zacherl MJ, Gildehaus FJ, Mittlmeier L (2020). First clinical results for PSMA targeted alpha therapy using ^225^Ac-PSMA-I&T in advanced mCRPC patients. J Nucl Med.

[25] Kratochwil C, Bruchertseifer F, Giesel FL (2016). ^225^Ac-PSMA-617 for PSMA-Targeted α-Radiation Therapy of Metastatic Castration-Resistant Prostate Cancer. J Nucl Med.

[26] Fendler WP, Eiber M, Beheshti M (2017). ^68^Ga-PSMA PET/CT: Joint EANM and SNMMI procedure guideline for prostate cancer imaging: version 1.0. Eur J Nucl Med Mol Imaging.

[27] Ferdinandus J, Violet J, Sandhu S (2020). Prognostic biomarkers in men with metastatic castration-resistant prostate cancer receiving [^177^Lu]-PSMA-617. Eur J Nucl Med Mol Imaging.

[28] Boellaard R, Delgado-Bolton R, Oyen WJG (2015). FDG PET/CT: EANM procedure guidelines for tumour imaging: version 2.0. Eur J Nucl Med Mol Imaging.

[29] Rosar F, Dewes S, Ries M (2020). New insights in the paradigm of upregulation of tumoral PSMA expression by androgen receptor blockade: Enzalutamide induces PSMA upregulation in castration-resistant prostate cancer even in patients having previously progressed on enzalutamide. Eur J Nucl Med Mol Imaging.

[30] Grubmüller B, Rasul S, Baltzer P (2020). Response assessment using [^68^Ga]Ga-PSMA ligand PET in patients undergoing systemic therapy for metastatic castration-resistant prostate cancer. The Prostate.

[31] Grubmüller B, Senn D, Kramer G (2019). Response assessment using 68Ga-PSMA ligand PET in patients undergoing ^177^Lu-PSMA radioligand therapy for metastatic castration-resistant prostate cancer. Eur J Nucl Med Mol Imaging.

[32] Wahl RL, Jacene H, Kasamon Y, Lodge MA (2009). From RECIST to PERCIST: Evolving Considerations for PET response criteria in solid tumors. J Nucl Med.

[33] Scher HI, Morris MJ, Stadler WM (2016). Trial Design and Objectives for Castration-Resistant Prostate Cancer: Updated Recommendations From the Prostate Cancer Clinical Trials Working Group 3. J Clin Oncol.

[34] Hartrampf PE, Heinrich M, Seitz AK (2020). Metabolic Tumour Volume from PSMA PET/CT Scans of Prostate Cancer Patients during Chemotherapy—Do Different Software Solutions Deliver Comparable Results?. J Clin Med.

[35] Michalski K, Mix M, Meyer PT, Ruf J (2019). Determination of whole-body tumour burden on [^68^Ga]PSMA-11 PET/CT for response assessment of [^177^Lu]PSMA-617 radioligand therapy: a retrospective analysis of serum PSA level and imaging derived parameters before and after two cycles of therapy. Nuklearmedizin.

[36] Schmuck S, von Klot CA, Henkenberens C (2017). Initial Experience with Volumetric ^68^Ga-PSMA I&T PET/CT for Assessment of Whole-Body Tumor Burden as a Quantitative Imaging Biomarker in Patients with Prostate Cancer. J Nucl Med.

[37] Schmidkonz C, Cordes M, Schmidt D (2018). ^68^Ga-PSMA-11 PET/CT-derived metabolic parameters for determination of whole-body tumor burden and treatment response in prostate cancer. Eur J Nucl Med Mol Imaging.

